# Intraspecific and Intrageneric Genomic Variation across Three *Sedum* Species (Crassulaceae): A Plastomic Perspective

**DOI:** 10.3390/genes15040444

**Published:** 2024-03-31

**Authors:** Sijia Zhang, Shiyun Han, De Bi, Jianke Yang, Wen Ge, Yuanxin Ye, Jinming Gao, Chenwei Dai, Xianzhao Kan

**Affiliations:** 1 Anhui Provincial Key Laboratory of the Conservation and Exploitation of Biological Resources, Wuhu 241000, China; zhangsijia@ahnu.edu.cn (S.Z.); hansy@ahnu.edu.cn (S.H.); ajiankebc@wnmc.edu.cn (J.Y.); yuanxye@ahnu.edu.cn (Y.Y.); gaojming@ahnu.edu.cn (J.G.); 2 Suzhou Polytechnic Institute of Agriculture, Suzhou 215000, China; bid@szai.edu.cn; 3 School of Basic Medical Sciences, Wannan Medical College, Wuhu 241002, China; 4 School of Food and Bioengineering, Wuhu Institute of Technology, Wuhu 241003, China; gwen@whit.edu.cn; 5 Anhui Academy of Medical Sciences, Anhui Medical College, Hefei 230061, China; 6 The Institute of Bioinformatics, College of Life Sciences, Anhui Normal University, Wuhu 241000, China

**Keywords:** *Sedum*, chloroplast genome, plastome variation, codon aversion, codon usage bias, non-monophyly, intraspecific level

## Abstract

*Sedum* is the largest succulent genus in Crassulaceae. Because of predominant maternal inheritance, little recombination, and slow evolution, plastomes can serve as powerful super barcodes for inter- or intra-species phylogenetic analyses. While previous research has focused on plastomes between *Sedum* species, intra-species studies are scarce. Here, we sequenced plastomes from three *Sedum* species (*Sedum alfredii*, *Sedum plumbizincicola*, and *Sedum japonicum*) to understand their evolutionary relationships and plastome structural evolution. Our analyses revealed minimal size and GC content variation across species. However, gene distribution at IR boundaries, repeat structures, and codon usage patterns showed diversity at both inter-specific and intra-specific levels. Notably, an *rps19* gene expansion and a bias toward A/T-ending codons were observed. Codon aversion motifs also varied, potentially serving as markers for future studies. Phylogenetic analyses confirmed the non-monophyly of *Sedum* and divided the *Acre* clade into two groups. Individuals from the same species clustered together, with strong support for the relationships between *S. alfredii*, *S. tricarpum*, and *S. plumbizincicola*. Additionally, *S. japonicum* clearly affiliates with the *Acre* clade. This study provides valuable insights into both intra-specific and intra-generic plastome variation in *Sedum*, as well as overall plastome evolution within the genus.

## 1. Introduction

*Sedum*, the largest genus of the family Crassulaceae, is a widely cultivated and diverse ornamental plant used in horticulture. It has various applications, such as those in medicine, ecology, and landscapes [1,2]. One of the most notable applications of the genus is a roof garden [3,4], which provides several benefits, including air filtration [5], temperature regulation [6], rainwater detention [7], and noise reduction [8]. According to World Flora Online (http://www.worldfloraonline.org/, accessed 1 February 2024), the genus *Sedum* currently consists of 476 accepted species. However, the phylogeny within the genus remains poorly understood and complex, and *Sedum* has been confirmed as non-monophyletic in previous studies [9,10,11]. Crassulaceae, also known as the stonecrop family, is a large family of succulent plants that contains over 1400 species across 33 genera. This family contains three subfamilies (Crassuloideae, Kalanchoideae, and Sempervivoideae), and the subfamily Sempervivoideae includes five clades, i.e., *Telephium*, *Sempervivum*, *Aeonium*, *Leucosedum*, and *Acre*. With approximately 73% of species assigned within the *Acre* clade, the remaining *Sedum* species are surprisingly intermingled with other Sempervivoideae clades [11]. This confusion may be due to insufficient data, limited resolution, or weak branch support [11,12,13]. To clarify the phylogenetic relationships within *Sedum*, further sampling and improved classification methods are needed.

With the advent of next-generation sequencing technology and the development of genome assembly software, plastomes are becoming easier to obtain. Because of maternal inheritance, little recombination, and slow evolution, plastomes can serve as powerful super barcodes for inter- or intra-species phylogenetic analyses [14,15,16]. Since the pioneering work by Shinozaki et al. [17] in reporting the first tobacco plastome, about 30,000 plastomes are now publicly available in NCBI (as of February 2024). Typically, these plastomes of higher plants can split into four distinct regions—large and small single-copy regions (LSCs and SSCs) and two inverted repeats (IRa and IRb), 120–160 kb in size [3]. These regions contain key genes responsible for photosynthesis and other metabolic functions. Furthermore, the IR regions are particularly important for maintaining the stability of the plastid genome and facilitating homologous recombination events. Moreover, the IR regions possess a distinct sequence composition that enables them to interact complementarily with one another. The typical plastome in the family Crassulaceae has a typical quadripartite structure, and it consists of 85 protein-coding genes (PCGs), 36–37 tRNA genes, 8 rRNA genes, and 4 pseudogenes. Therefore, investigating the structure and gene composition of plastomes is necessary for conducting comparative genomic studies and unraveling the mechanisms of plastid genome evolution.

While inter-species plastomic analyses have been conducted on various Crassulaceae genera (e.g., *Crassula* [10], *Aeonium* and *Monanthes* [18], *Kalanchoe* [19], *Rhodiola* [20,21,22], *Telephium* clade [16], and others [23,24,25]), research on intra-species diversity within Crassulaceae plastomes is lacking. Within a species, intra-species diversity describes the genetic differences found among individuals. The analysis of intra-species diversity can offer valuable insights into both evolutionary adaption and biodiversity conservation. Accordingly, further investigation in this field is required.

Codon usage bias (CUB), a phenomenon where synonymous codons (those coding for the same amino acid) are used unevenly within a genome, offers a powerful tool to investigate the evolutionary history of both taxa and genes [26,27]. The examination of CUB can offer valuable insights into genome evolution, including mutational preferences, translation efficiency, and the impact of selective pressures on various genes and genomic regions. Relative synonymous codon usage (RSCU) values are determined by dividing the observed occurrence of a codon by its expected frequency of that codon. They are used to quantify codon usage bias, which is the non-random use of synonymous codons. By examining RSCU patterns and codon preferences, we can infer the molecular mechanisms that influence codon usage, which may vary between various organisms, tissues, or developmental stages. Additionally, codon aversion motifs (CAMs), presented as the nonuse of codons in genes, have recently been proposed to be a novel marker for phylogenetic studies [16,18,28]. Compared to traditional alignment-based techniques, this CAM-based approach has several advantages, such as quicker computation and reduced complexity.

In this study, we present newly sequenced plastomes from sixteen individuals of three *Sedum* species (*S. alfredii*, *S. plumbizincicola*, and *S. japonicum*) belonging to the *Acre* clade. By combining these new data with existing plastome sequences retrieved from NCBI, we aim to address the following: (1) intraspecific and intrageneric plastomic variation across the three species and (2) the phylogeny of the *Acre* clade. Our findings provide valuable insights into both intraspecific and intrageneric plastome variation in *Sedum*, as well as overall plastome evolution within the genus.

## 2. Materials and Methods

### 2.1. Sample Collection and Plastome Sequencing

Sixteen samples from three *Sedum* species were collected in Eastern China, with five each from *S. alfredii* and *S. japonicum* and six from *S. plumbizincicola*. This ensured a good representation of the genetic variation within each species. Total DNA was extracted using the CTAB method [29]. In this approach, cetyltrimethylammonium bromide was employed to lyse cells, followed by the addition of organic solvents and alcohol precipitation to isolate DNA. In the next step, we used the TruSeq^®^ DNA PCR-Free Library Prep Kit [30] to create a sequencing library, which is specifically designed to produce libraries that are high-quality and uniform. The resulting library was then sequenced on the Illumina Hiseq X Ten [31]. This is a high-throughput sequencing platform that allows for the parallel sequencing of millions of DNA fragments simultaneously. For this sequencing run, 150 paired ends and a 350 bp insert size were employed. Paired-end reads provide more accurate sequence data compared to single-end reads.

### 2.2. Plastome Assembly, Annotation, and Nucleotide Compositions Analysis

For this study, a multi-step process was used to examine the plastome sequences of the collected three Sedum species. This process included assembly, annotation, and comparative analysis. In the beginning, raw sequencing data were processed and assembled using GetOrganelle 1.7.5 [32], a pipeline created especially for the efficient assembly of organelle genomes from whole-genome sequencing data. The automated annotation program GeSeq [33], which identifies protein-coding and tRNA and rRNA genes in chloroplast genomes, was then used to annotate the genomes. The plastomes of *S. sarmentosum* (NC_023085), *S. oryzifolium* (NC_027837), and *S. lineare* (NC_052707) served as references, guaranteeing precise gene identification and boundary determination. In order to improve annotation results, manual correction was then carried out.

For further analysis, the only available plastome sequence of *S. alfredii* (NC_064359) was selected, providing additional context and a valuable basis for comparison. Each plastome was drawn individually using Chloroplot [34], a visualization tool that generates graphical representations of the circular chloroplast genome structure.

To better understand the structural features and junction sites of the plastome sequence, IRscope [35] was employed. This tool visualizes the features of genome structure, illustrating the proportional representation of plastome sequences on their respective junction sites. Nucleotide composition analysis was performed to assess the relative abundance of each nucleotide in the plastome sequences. This provided insights into the genomic composition and potential functional implications of the observed patterns.

### 2.3. Analyses of Dispersed and Simple Sequence Repeats

The online programs REPuter [36] and Misa [37] were used for identifying and comparing repeat sequences among the three species. These tools allowed for a comprehensive exploration of dispersed and simple sequence repeats, providing useful insights into the genomic composition. REPuter was used to search for dispersed repeats, with the following settings: all types of match direction, a hamming distance of 3, and a size limit of 30 to 300 bp. The hamming distance parameter allowed for a certain degree of sequence variation in the detected repeats, while the size limit ensured the identification of both short and moderately-sized dispersed repeats. By encompassing various match directions, this analysis captured a wide range of potential dispersed repeats across the genomes. In addition to dispersed repeats, Misa was employed to predict the number of simple sequence repeats (SSRs) within the examined genomes. The default search parameters were used, which include six groups of motif lengths (mono- to hexa-nucleotide motifs), a minimum number of repetitions, and a maximum interval length of 100 bp.

### 2.4. Codon Usage Analysis of the Plastome

To investigate the RSCU of the plastome, CodonW v.1.4.4 [38] was applied to calculate the values, excluding the termination codons. The RSCU is a value calculated to judge the usage frequency of different codons coding the same amino acid with the equation [39] as follows:

$$RSCU_{ij}=\frac{x_{ij}}{\frac{1}{n_{i}}}\sum_{j=1}^{n_{i}}x_{i_{j}}$$

A codon usage frequency value greater than 1 indicates that the codon is used more often than the typical usage of the synonymous codons. Conversely, a value less than 1 indicates the opposite. Additionally, a heatmap was generated using the software TBtools [40] to visualize the codon usage frequencies with color shades. This visualization allows for a quick and easy identification of codons with biased usage patterns. Furthermore, CAM [28] was conducted to explore codon aversion in coding DNA sequence (CDS) with a length over 300 bp among the 18 plastomes.

### 2.5. Phylogenetic Analysis

To better understand the relationships among the three *Sedum* species, we conducted a phylogenetic analysis within the *Acre* clade of Sempervivoideae, using *Rosularia alpestris* as the outgroup (Table S1). A total of 36 plastomes were used, and 79 CDS were extracted from each. The multiple sequences were aligned by MAFFT 7.520 [41] to obtain an aligned CDS dataset, which was then concatenated using PhyloSuite 1.2.3 [42]. Both maximum likelihood (ML) and Bayesian inference (BI) methods were performed to reconstruct evolutionary trees.

For the ML analysis, RAxML 8.0.0 [43] was employed based on the concatenated sequences, using 1000 bootstrap replicates and the GTRCAT model. Convergence was checked under the autoMRE criteria. For the BI inference, the processing files were created by SequenceMatrix 1.9 [44], and the best model was selected by ModelTest-NG 0.1.7 [45] among the split CDSs. MrBayes 3.2.7 [46] was then run for 10 million generations, sampling every 1000th generation. All Markov chain Monte Carlo (MCMC) simulations were repeated twice, and Tracer v.1.7.1 [47] was used to verify convergence.

## 3. Results

### 3.1. Comparative Analysis of Composition and Structure

**Figure 1:**
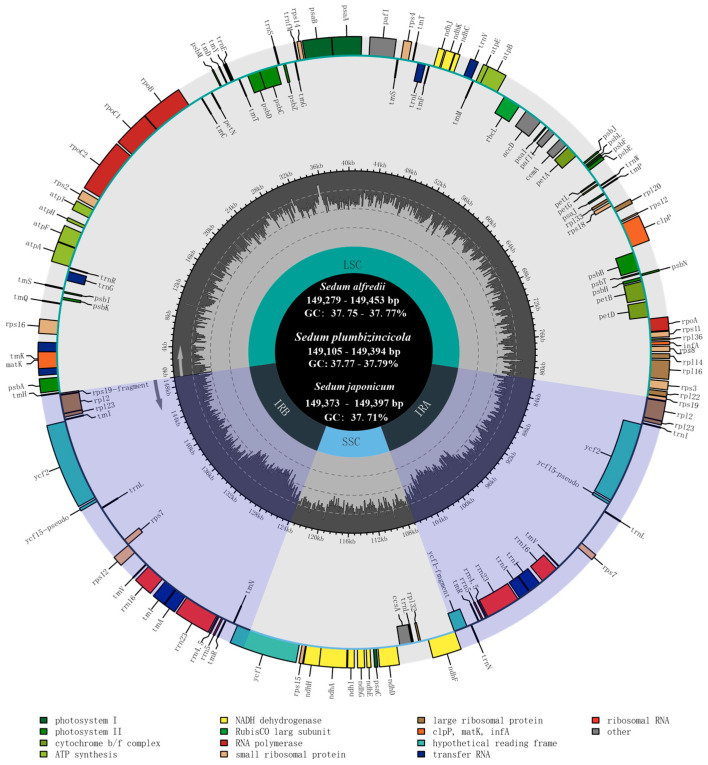
Plastome annotation map for 18 individuals of *S. alfredii*, *S. japonicum*, and *S. plumbizincicola*. Genes within the circle are transcribed clockwise, while outside genes are counterclockwise. Dark gray and light gray regions in the inner ring showed the ratio of G+C and A+T.

Based on the assembled 16 plastomes, the estimated read coverage all reached 100% (Table S2, Supplementary Materials). Analysis of plastome size and GC content revealed no significant variations among the three investigated *Sedum* species. The length of the sequences ranged from 149,105 to 149,394 bp in *S. plumbizincicola*, with a maximal difference of 289 bp. In *S. alfredii*, the length ranged from 149,279 to 149,453 bp, while in *S. japonicum,* the range was 149,373 to 149,397 bp, with a minimum difference of 24 bp. Notably, the GC content for all individuals across the three species was highly similar, with a narrow range of 37.7% to 37.8% (Figure 1, Table 1).

**Figure 2:**
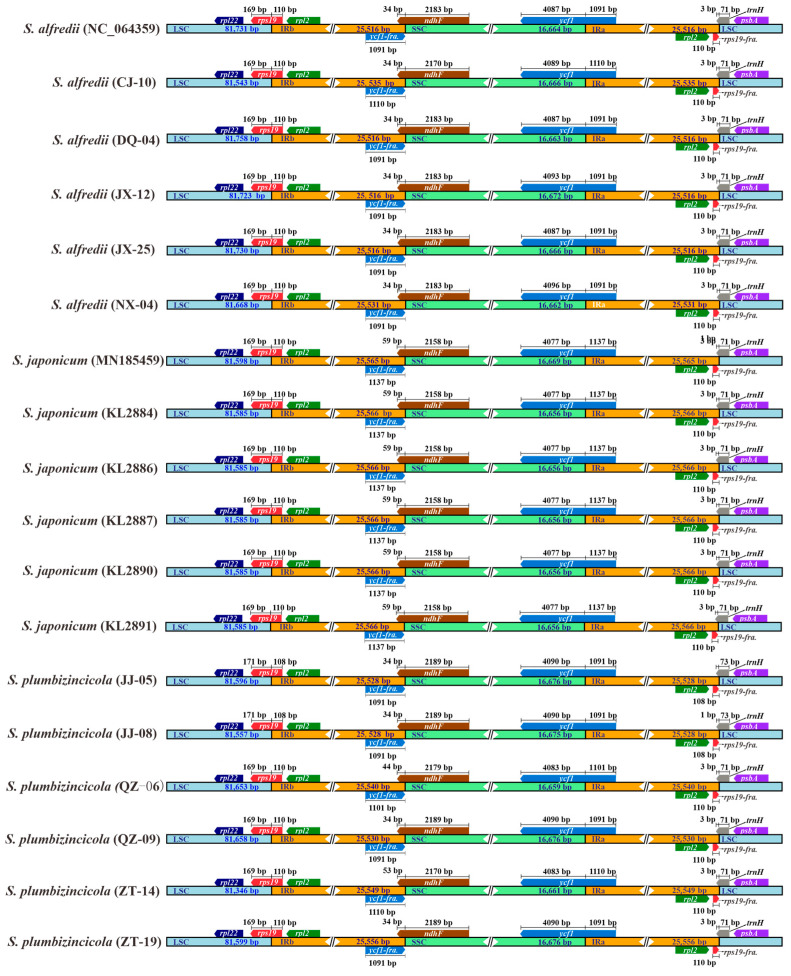
Comparative analysis of IR junctions among 18 individuals focused on gene length. IRa and IRb are reciprocal inverted repeat sequences. LSC is the abbreviation for long single copy, and SSC means short single copy. Different colors are used to represent different regions and genes, and “fra” represents fragment.

Comparative analysis of all plastomes of the three species revealed a conserved model within the IR regions and the gene structures flanking the four distinct junction sites. The genes *rps19*, *ndhF*, *ycf1*, and *trnH*, located at the IR junctions, share a portion of their sequence with the inverted repeats of all the plastomes. Additionally, our study demonstrates a consistent gene order and a high degree of similarity in the length of IR regions. However, some contrasts still exist in the IR-boundary distribution of genes between species and even within the same species. For example, *S. plumbizincicola* exhibits the highest length variations across all genes or regions, as shown in Figure 1. Conversely, *S. japonicum* displays the lowest variations based on standard deviation (StDev) calculations (Table 1). Furthermore, the length of the *ndhF* gene extending into the IRb region can be used to distinguish *S. japonicum* from the other two species. For all *S. japonicum* samples, their lengths reach 59 bp, while those of the other two species are 34, 44, or 53 bp. The extended lengths of the samples in *S. plumbizincicola* fall in between with variations (Figure 2, Table S3).

### 3.2. Analysis of Repeats in Three Sedum Species

**Figure 3:**
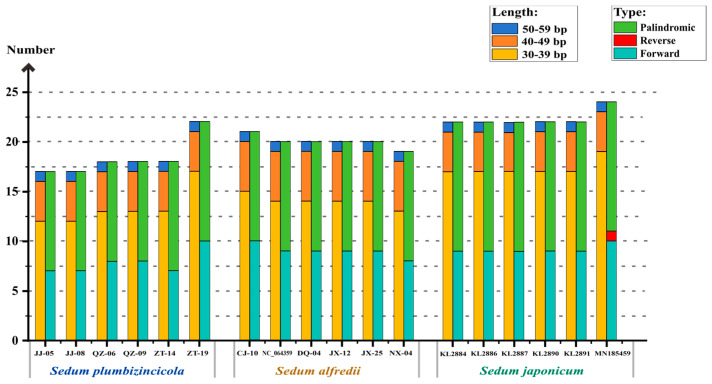
Dispersed repeats in *S. plumbizincicola*, *S. alfredii*, and *S. japonicum*, calculated for different length regions (**left**) and repeat types (**right**) for a total of 18 individuals.

This study identified 110, 120, and 134 dispersed repeat members in *S. plumbizincicola*, *S. alfredii*, and *S. japonicum*, respectively. We analyzed these repeats based on both type and length (Figure 3, Table S4). The results showed that most repeats were 30–39 bp in length, forward, and palindromic, which is the main source of variation between the species. Notably, the number of repeats with 40–49 bp and 50–59 bp remained stable across all three species. Interestingly, the 40–49 bp repeats even can be used to distinguish *S. alfredii* because of the unique presence of five such repeats. Furthermore, reverse dispersed repeats were observed in *S. japonicum*, but with very low probability (only one instance in an individual).

**Figure 4:**
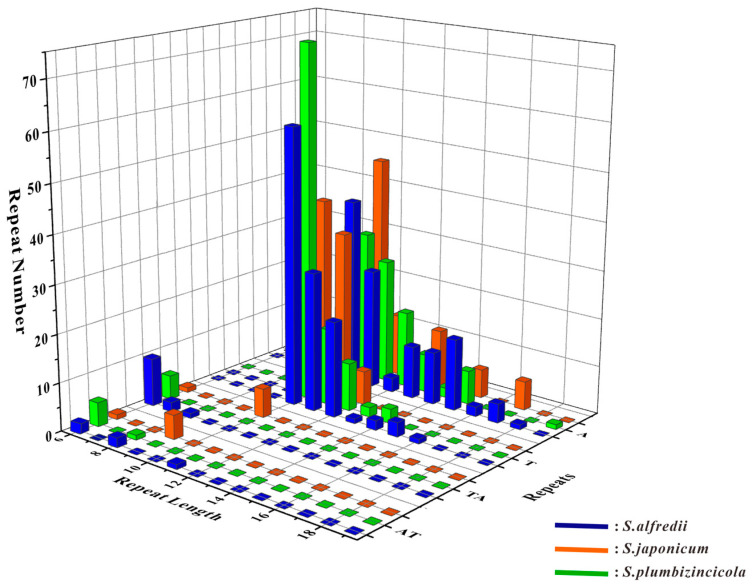
Statistic of simple sequence repeats in *S. alfredii*, *S. japonicum*, and *S. plumbizincicola*. The coordinate system is built with repeat length as the X-axis, repeat types as the Y-axis, and the number of repeats as the Z-axis. Different colors represent different species in the 3D bar chart.

For the SSR analysis, only mononucleotides and dinucleotides repeats were discovered in the three *Sedum* species. These species shared the same types of SSRs: A, AT, T, and TA. Most of these SSRs were mononucleotides (A/T), covering over 90% of all identified repeats. However, some variations in number were observed. *S. japonicum* had the least number of SSRs, with only 180, while *S. alfredii* had the most, with 243 (Table S5). The distributions of these SSRs showed relatively distinct differences, as illustrated in Figure 4.

### 3.3. Codon Usage and Aversion Patterns

**Figure 5:**
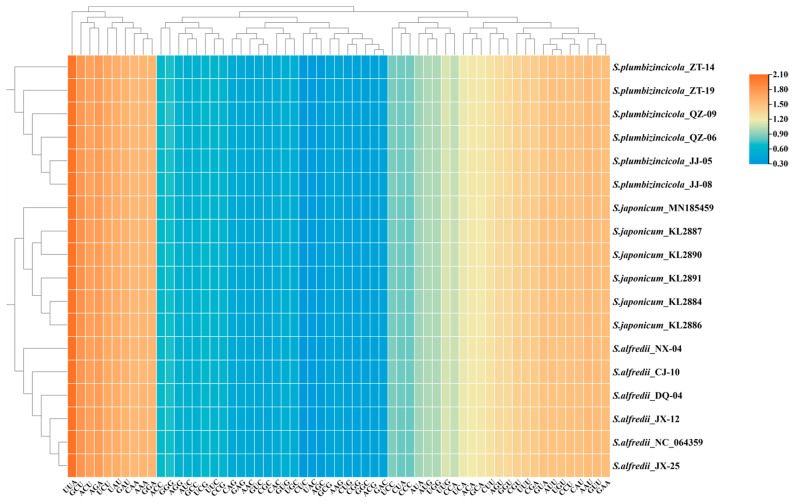
Heat map of 53 CDSs (length > 300 bp) concatenated together for 18 plastomes. The colors change with the frequency of codon usage. A value of 1 is the boundary of bias (positive bias if the value > 1 and vice versa).

A total of 53 genes were investigated for codon usage and aversion. In our study, all RSCU values exhibited a highly similar pattern among the 18 plastomes, with the lowest values (0.32) observed for CUC and the highest value (2.07) found for UUA (Figure 5, Table S6). Interestingly, both extreme values occurred for the same amino acid, leucine. This suggests a strong codon bias for leucine in these plastomes. Six codons were overrepresented (RSCU > 1.6), while 21 codons were underrepresented (RSCU < 0.6). Notably, a significant preference for A/T-ending codons was observed across all species. Variations in RSCU values (Table S6) existed at the intraspecies level. In *S. plumbizincicola*, only 22 of 61 (excluding stop codons) had the same RSCU values as the average across all individuals. *S. alfredii* showed even fewer consistent codons (16). Conversely, *S. japonicum* exhibited consistency in most codons (59).

**Figure 6:**
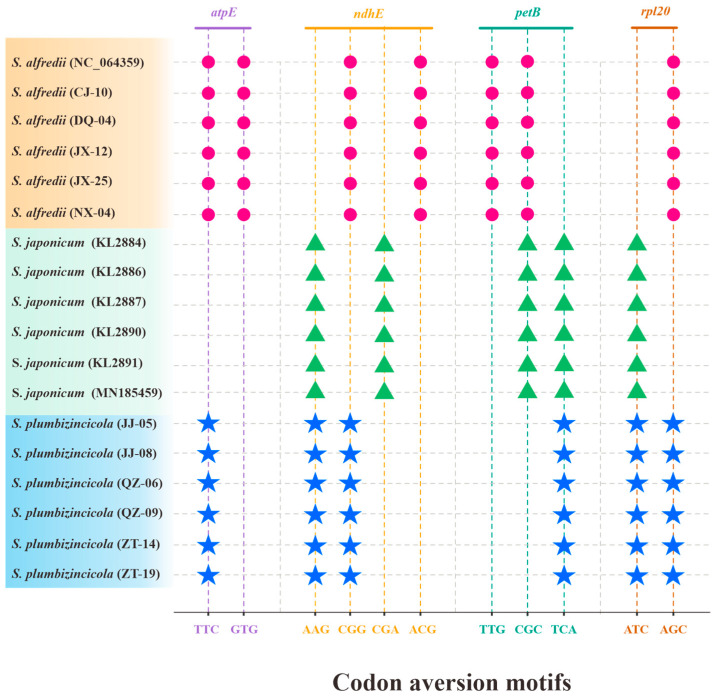
Species-specific codon aversion motifs of the three *Sedum* species. The same color of imaginary lines and codons reflects that they are in the same gene, and the dots with the same shape and color represent the same species.

Regarding codon aversion motifs, our work revealed both similarities and variations across the three species. Aversion patterns were present in most of the genes, with only five exceptions: *rpoB*, *rpoC1*, *rpoC2*, *ycf1,* and *ycf2*. Notably, these patterns could effectively distinguish the species. Four genes (*atpE*, *ndhE*, *petB*, and *rpl20*) completely separated the three species (Figure 6), while an additional 11 genes differentiated *S. alfredii* and *S. plumbizincicola* (Table S7). Similar to RSCU values, individual variations were also observed in codon aversion motifs. Thirteen genes displayed individual-specific aversion patterns, with 10 in *S. plumbizincicola*, 6 in *S. alfredii*, and none in *S. japonicum*. Interestingly, *matK*, *ndhJ*, and *rps3* genes exhibited variations in both *S. plumbizincicola* and *S. alfredii* (Table S8).

### 3.4. Phylogenetic Analysis

**Figure 7:**
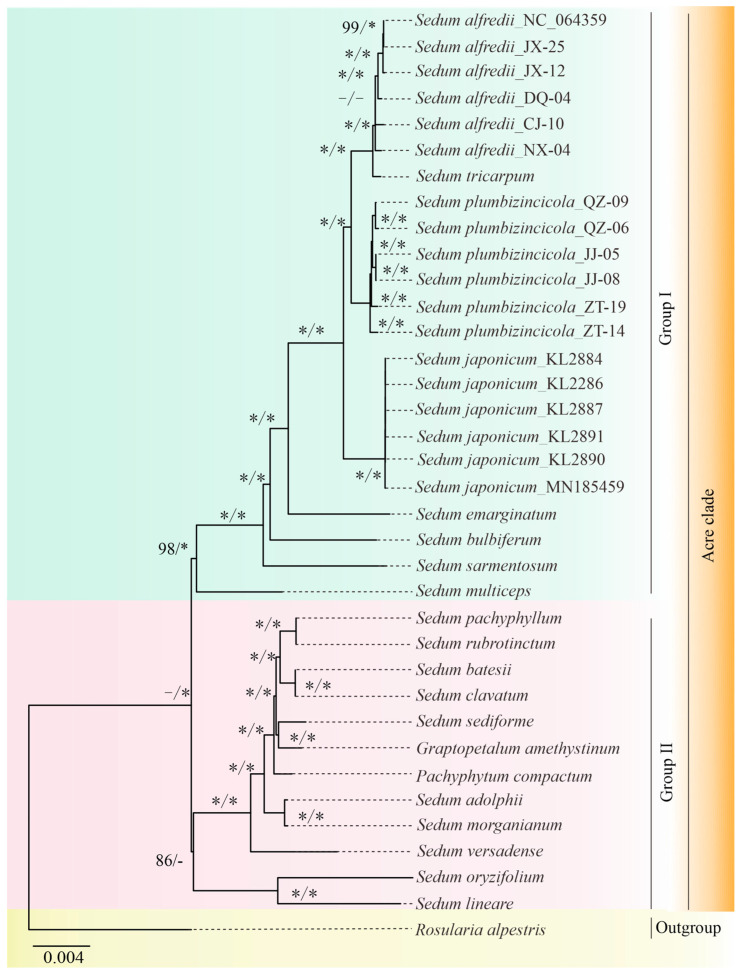
Phylogenetic tree for 36 plastomes, including 35 plastomes in the *Acre* clade and 1 in the *Leucosedum* clade as the outgroup, using both ML and BI methods. The values on the left are [BS], while those on the right represent [pp]. The symbol “−“ denotes low support values or the lack of the values, and “*” represents 100 or 1.00 support values.

The ML and BI inferences were obtained using a 68,382-bpPCG matrix. Our ML analysis revealed that the genus *Sedum* is non-monophyletic, and the *Acre* clade could be further divided into two groups (Figure 7). Group I contains 23 plastomes from seven species ([BS] = 98), all belonging to *Sedum*. The remaining 12 plastomes belong to Group II ([BS] = 86), comprising individuals from three genera: *Sedum*, *Pachyphytum,* and *Graptopetalum*. Notably, Group II lacked support from our BI analysis.

Although the tree topologies constructed by the two methods were slightly different, our phylogenetic analyses successfully inferred both inter- and intra-specific relationships within and among the three studied *Sedum* species (*S. japonicum*, *S. alfredii*, and *S. plumbizincicola*), except for *S. japonicum* specimen. Individuals from the same species were grouped, and all eighteen individuals formed distinct clusters with high support in both trees with strong support values ([BS] = 100, [PP] = 1.00). Furthermore, *S. alfredii* and *S. tricarpum* share a close relationship and form a sister group with *S. plumbizincicola* ([BS] = 100, [PP] = 1.00). These three species, along with *S. japonicum*, together constitute a clade ([BS] = 100, [PP] = 1.00).

## 4. Discussion

In this study, we report the plastome sequences of *S. japonicum* for the first time and augment the plastome data for *S. alfredii* and *S. plumbizincicola*. In total, we contribute 16 new plastome datasets, addressing the data gap [11] on *Sedum* and providing additional molecular evidence for species identification. Through comparative analysis of sequence composition, structure, and codon usage bias, we surprisingly found diversities at both interspecific and intraspecific levels.

The expansion and contraction events in IR regions are common in plastomes and are closely related to the evolutionary history of species [48]. For instance, the 110 bp expansion of the *rps19* gene was identified as a specific marker because of its universality in *Aeonium*, *Monanthes*, and some other genera [10,18]. While most sequences share this feature, there are still exceptions in *S. plumbizincicola* individuals, with lengths of 108 and 109 bp. Therefore, we propose that this approximately 110 bp expansion can serve as a potential marker for phylogenetic studies within the Crassulaceae family.

Mononucleotide SSRs are consistently abundant in plastomes [49,50], with some studies reporting them as the most common type of SSR [51]. Among these mononucleotide repeats, while C/G type SSRs may be prevalent in specific species [5,52], A/T types are more commonly observed in land plants [53,54,55,56,57]. Our research confirms this pattern, showing an A/T proportion of over 90%, significantly exceeding other types. Furthermore, because of their high diversity, SSRs are widely used to study phylogenetic relationships, genetic variation, and evolutionary mechanisms [58]. Though variability in SSR numbers is common above genus level (minimal among close relatives) [51], our study of three *Sedum* species reveals detectable differences in number, length, and motif distribution.

Like other Crassulaceae species [10,16,18], A/T-ending codons are favored in *S. alfredii*, *S. japonicum*, and *S. plumbizincicola*. As previous studies have shown, a close association exists between RSCU values and species phylogeny [59,60,61]. Consistent with this, our heatmap analysis reveals three distinct clusters, reflecting a closer relationship between *S. japonicum* and *S. alfredii*. This further supports the notion that RSCU may harbor considerable phylogenetic implications. However, this finding contradicts our phylogenetic results, which need further exploration.

To date, research on specific codon aversion analysis remains limited. However, several studies have demonstrated the effectiveness of codon aversion motifs in distinguishing different lineages based on specific characteristics [9,16,18]. Interestingly, our investigation focused on the species level and revealed that individuals within the same species can exhibit diverse codon aversions.

Despite significant morphological similarities, heavy metal enrichment capacity, and some molecular features, *S. alfredii* and *S. plumbizincicola* exhibit distinct characteristics [62,63,64,65]. This study provides more specific molecular evidence to support their independent status. Phylogenetic trees clearly separate the 12 individuals into three lineages, with *S. tricarpum* and *S. alfredii* forming a sister clade to *S. plumbizincicola*. Furthermore, *S. japonicum*, whose plastome sequences were first reported in this study, showed high similarities to *S. alfredii* and *S. plumbizincicola* in nucleotide composition, structure, and codon usage motifs. Moreover, phylogenetic trees consistently place S. *japonicum* within a clade containing *S. alfredii*, *S. plumbizincicola*, and *S. tricarpum*, with strong statistical support. This suggests a potential affiliation of *S. japonicum* is assigned to the *Acre* clade.

## Figures and Tables

**Table 1:** The sample information and genomic features of the investigated species.

Species	Source/Voucher	Accession Number	Locality	GC Content (%)	Length (bp)
***S. alfredii***	CJ-10	PP234500 *	Chunjian, Hangzhou, China	37.77	149,279
(149,401 ± 63 bp)	DQ-04	PP234501 *	Dongqiao, Hangzhou, China	37.75	149,453
	JX-12	PP234502 *	Jiuxi, Hangzhou, China	37.76	149,427
	JX-25	PP234503 *	Jiuxi, Hangzhou, China	37.76	149,428
	NX-04	PP234504 *	Ningxi, Taizhou, China	37.77	149,392
	Shaocui et al. (2022)	NC_064359	Daqiao, Hangzhou, China	37.76	149,427
***S. japonicum***	KL2884	PP234494 *	Dashan, Chizhou, China	37.71	149,373
(149,377 ± 10 bp)	KL2886	PP234495 *	Dashan, Chizhou, China	37.71	149,373
	KL2887	PP234496 *	Dashan, Chizhou, China	37.71	149,373
	KL2890	PP234497 *	Dashan, Chizhou, China	37.71	149,373
	KL2891	PP234498 *	Dashan, Chizhou, China	37.71	149,373
	Ding et al. (2019)	MN185459	Dashan, Chizhou, China	37.71	149,397
***S. plumbizincicola***	JJ-05	PP234493 *	Jiangjia, Hangzhou, China	37.77	149,328
(149,316 ± 112 bp)	JJ-08	PP234488 *	Jiangjia, Hangzhou, China	37.77	149,288
	QZ-06	PP234489 *	Shangfang, Quzhou, China	37.78	149,392
	QZ-09	PP234490 *	Shangfang, Quzhou, China	37.77	149,394
	ZT-14	PP234491 *	Zitong, Hangzhou, China	37.79	149,105
	ZT-19	PP234492 *	Zitong, Hangzhou, China	37.78	149,387

* These new plastomes were generated in this study.

## Data Availability

The 16 plastomes sequences data generated in this study are available in GenBank of the National Center for Biotechnology Information (NCBI) (https://www.ncbi.nlm.nih.gov/nuccore accessed 1 February 2024) under the access numbers: PP234488–PP234498 and PP234500–PP234504.

## Supplementary Materials

The following supporting information can be downloaded at https://www.mdpi.com/article/10.3390/genes15040444/s1: Table S1: Additional species included for phylogenetic tree; Table S2: Reads coverage and depth of 16 Sedum samples that are mapped to the assembled plastomes; Table S3: Sizes of plastomic quadripartite regions and boundary-located genes in three *Sedum* species; Table S4: Distributions of the dispersed repeats classified by type and length; Table S5: Distributions of the SSRs classified by type and length; Table S6: RSCU values of 18 investigated plastomes; Table S7: Specific codon aversion motifs only separate *S. alfredii* and *S. plumbizincicola*; Table S8: Variable codon aversion motifs at intra-specific level.
